# Choroid plexus tuberculoma: Diagnosis, management and role of endoscopy

**DOI:** 10.17712/nsj.2017.3.20160465

**Published:** 2017-07

**Authors:** Nishanth Sadashiva, Bevinahalli N. Nandeesh, Dhaval Shukla, Bhagavatula I. Devi

**Affiliations:** *From the Department of Neurosurgery (Sadashiva, Shukla, Devi), and the Department of Neuropathology (Nandeesh), National Institute of Mental Health and Neurosciences, Bengaluru, India*

## Abstract

Ventricular involvement in central nervous system tuberculosis can be in the form of tuberculous ependymitis, intraventricular tuberculoma, intraventricular tuberculous abscess, choroid plexitis and choroid plexus tuberculoma. Only a few cases of choroid plexus tuberculomas have been described and even more rare is the description of the role of endoscopy in management of intraventricular tuberculomas. We report a 33-year-old patient while on treatment for tubercular meningitis who developed a left side choroid plexus lesion with loculated temporal horn. To confirm the diagnosis, endoscopic biopsy of the lesion was carried out. The final histopathology was tuberculoma. Intraventricular tuberculomas are usually associated with recalcitrant lesions, probably due to the poor drug levels within the CSF or as an indirect effect of immune resistance and biopsy becomes important to rule out other possibilities.

Ventricular involvement in cases of neurotuberculosis is rare. Only few cases with intraventricular tuberculomas are reported in the literature.^1^ Clinical features of patients with intraventricular tuberculomas are nonspecific and MRI plays a crucial role in diagnosis and monitoring treatment’s response.^2^,^3^ A few cases of intraventricular tuberculomas associated with parenchymal lesions, meningitis or ventriculitis are reported,^3^-^8^ but solitary intraventricular tuberculomas involving the choroid plexus are extremely rare and only 3 cases have been previously reported.^9^ There is paucity of literature concerning treatment aspects of neurotuberculosis with ventricular involvement.^3^ These lesions might be recalcitrant due to the poor drug levels within the cerebrospinal fluid (CSF) or as an indirect effect of immune resistance.^8^ Previous descriptions of treatment modalities for intraventricular tuberculoma included medical therapy in children and surgical excision of the lesion in adults. We describe a patient with tubercular meningitis who developed intraventricular lesion, and discuss the treatment aspects and peculiarities of ventricular involvement of neurotuberculosis.

## Case Report

**Figure 1:**
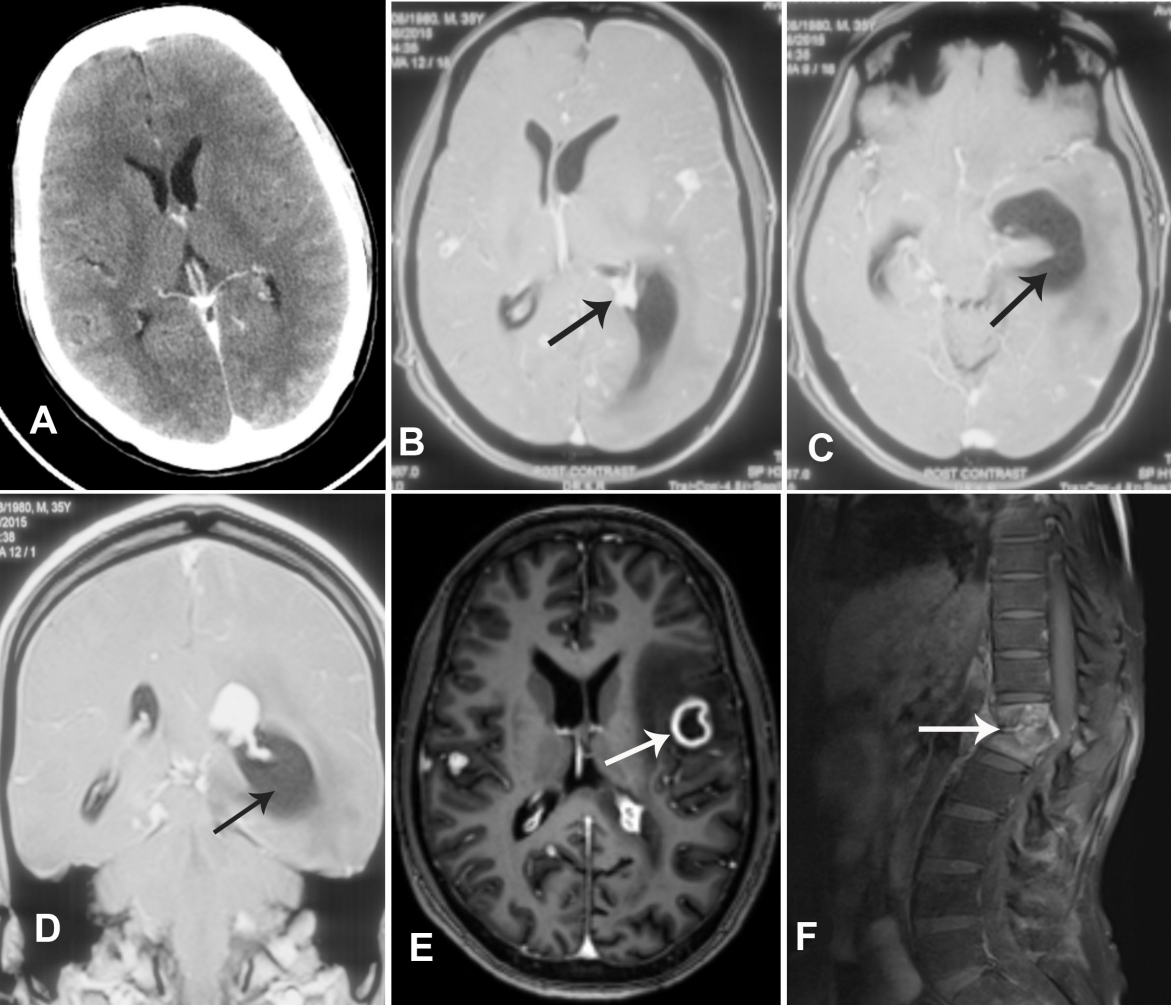
A computer Tomography carried out at **A)** diagnosis of tubercular did not show any lesion. **B)** Post gadolinium contrast enhanced MRI at time of presentation revealing a left atrial enhancing lesion causing loculated temporal horn (arrow shows). **C & D)** Loculated temporal horn caused by a lesion in left atrium of lateral ventricle (arrow shows). **E)** Post gadolinium contrast enhanced MRI revealing left frontal opercular ring enhancing lesion (arrow shows). **F)** MRI of spine showing D12-L1 vertebral collapse with para-spinal abscess enhancing on contrast. (arrow shows)

**Figure 2:**
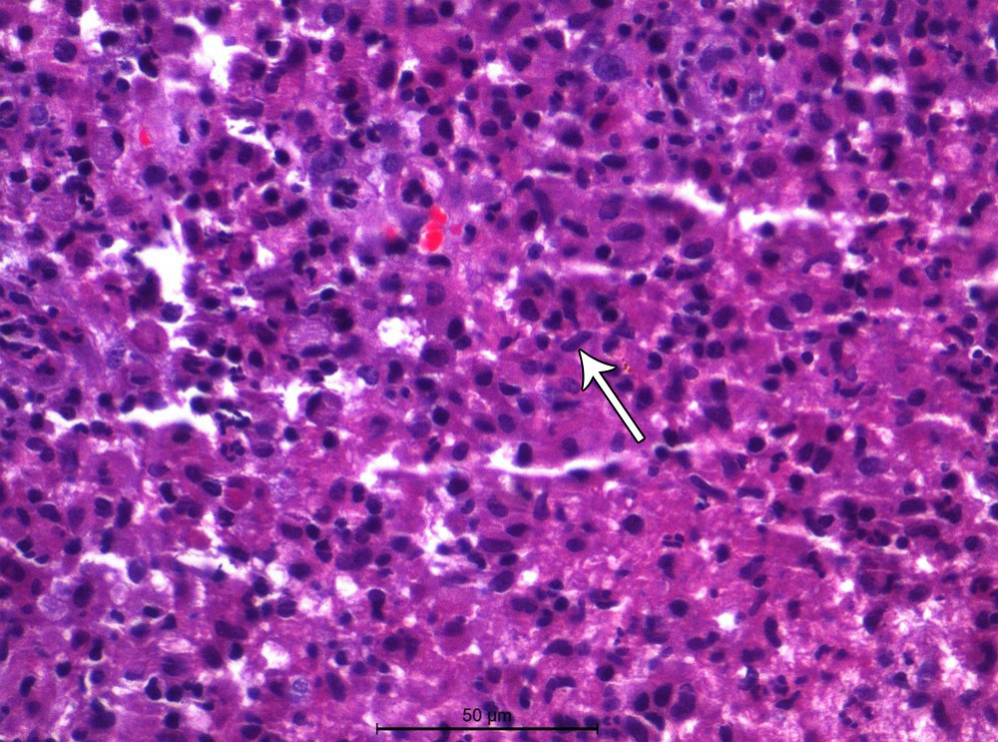
Microphotograph showing loose aggregates of epitheloid cells (arrow) admixed with histiocytes forming an ill-formed granuloma [H&E x 200] suggestive of tuberculoma.

A 33-year-old male had a history of headaches and intermittent fever. He was evaluated at a local hospital and diagnosed to have tubercular meningitis and started on anti-tubercular treatment (ATT) comprising Isoniazid 10mg/kg, Rifampicin 15mg/kg, Pyrazinamide 35mg/kg and Ethambutol 20mg/kg, 2 months prior. A head CT scan carried out at that time had not shown any mass lesion (**Figure 1A**). He became unconscious one week back, and was referred to our hospital. On arrival to casualty he was in altered sensorium, he was drowsy, obeying simple commands, and disoriented. The MRI showed multiple intracranial enhancing lesions suggestive of tuberculomas with a lesion in the left atrial region causing loculation of temporal horn and hydrocephalus (**Figure 1 B, C and D**) with mass effect on brain stem. A test to diagnose human immunodeficiency virus infection was negative. He initially underwent tapping of the temporal horn in emergency basis. As he was taking antitubercular drug therapy, and new lesions appeared, the possibility of drug resistant tuberculosis or alternate diagnosis was considered. A procedure that can be diagnostic and therapeutic was desirable, hence he underwent left temporal burr hole and through loculated temporal horn endoscopic biopsy of the lesion and thereby unblocking the loculated temporal horn. The lesion was white and firm in consistency diffusely involving the choroid plexus. The ventricular walls were looking normal. A biopsy was taken from the choroid plexus lesion and fenestration of loculated temporal was carried out connecting it to the lateral ventricles. Histopathological examination showed necrotizing granulomatous inflammation in the choroid plexus consistent with tuberculoma (**Figure 2**). Post operatively, he was continued on daily antitubercular treatment. About 40 days later, he lapsed into altered sensorium and was localizing to painful stimuli. Head CT scan showed re-loculation of temporal horn for which he underwent temporal horn-peritoneal shunt after which he improved. After about 4 months, he developed speech difficulty with righthemiparesis and ataxia. He had severe lower back pain with difficulty in walking. An MRI showed increase in size of caseating tuberculoma in left frontal opercular region (**Figure 1 E**) along with some resolution of choroid plexus lesion. There was D12-L1 vertebral osteomyelitis with paravertebral abscess (**Figure 1 F**). As patient was not willing to undergo surgery for spinal stabilization, he underwent CT guided biopsy and aspiration of para-spinal abscess. The microbiological examination of pus showed acid fast bacilli morphologically resembling mycobacterium tuberculosis complex on Ziehl-Neelsen (ZN) staining. He was asked to continue antitubercular drug therapy. At 16 months of follow-up from initial diagnosis and treatment he has arrest of progression of disease.

## Discussion

Ventricular involvement in central nervous system tuberculosis (CNS TB) is rare. It manifests in the form of tuberculous ependymitis, intraventricular tuberculoma, intraventricular tuberculous abscess, choroid plexitis, and choroid plexus tuberculoma.^1^ The literature describing ventricular tuberculosis as a complication of CNS TB is limited. Intraventricular tuberculoma can develop due to contiguous parenchymal tuberculoma extension, hematogenous seeding of the mycobacterium into the ependymal/choroid plexus forming a tuberculoma or rupture of these tubercular granulomas into the ventricles.^10^ Previously reported characteristic MRI findings of ventricular involvement in tuberculosis include contrast medium enhancement of the ependymal wall of the ventricle with hydrocephalus, intraventricular septations with sequestered ventricles, and ventricular sludge.^2^ Our patient had enhancing lesion in the choroid plexus without any other features of ventriculitis. Definitive diagnosis of tubercular ventriculitis requires demonstration of M. tuberculosis in the CSF and exclusion of meningitis/ ventriculitis like fungal and neoplasm. However, it is difficult due to a poor yield of M. tuberculosis by ZN staining of CSF, culture of CSF, or polymerase chain reaction.^1^

Although various authors have identified the presence of small tubercles in the choroid plexus, ependymal scarring, and thickened membrane as features observed within the ventricle following tuberculous infection,^4^ a well-defined intraventricular tuberculoma is a rare entity. Solitary intraventricular tuberculomas involving the choroid plexus are extremely rare and 3 cases have previously been reported.^9^ Even intraventricular tuberculomas associated with parenchymal lesions, meningitis or ventriculitis are also few.^3^-^8^ Non-Caseating tuberculomas are relatively hypointense on T1W imaging, hyperintense on T2W imaging, and show homogeneous enhancement after gadolinium administration. Caseating tuberculomas show rim enhancement on contrast-enhanced images.

Effective management protocol for intraventricular tuberculomas is unclear.^3^ This entity might be recalcitrant, probably due to the poor drug levels within the CSF or as an indirect effect of immune resistance.^8^ Previous descriptions of treatment modalities for intraventricular tuberculoma included medical therapy in children^4^ and surgical excision of the lesion in adults.^3^,^5^,^7^,^8^ Though medical management is accepted as standard treatment, adjunct treatments including endoscopic biopsy, surgical excision, and intraventricular drug therapy can be tried in case of diagnostic dilemmas, and relative resistance to standard therapy. Intraventricular tuberculomas with septations and large caseating tuberculomas require long-term treatment.^1^ Neurotuberculosis is quite common in India and biopsy is useful to exclude other conditions especially in non-responding patients and patients who are having paradoxical response. In a similar report by Udayakumaran et al^3^ endoscopic biopsy of a tuberculoma, and 3 surgical procedures were carried out for CSF diversion. Some studies have even demonstrated intrathecal injection of isoniazid and dexamethasone with multidrug chemotherapy as highly efficacious.^1^ The scenario demonstrates the complexity of the condition where multiple procedures are required and definitive treatment is not defined with treatment response being poor in intraventricular tuberculosis.

Our patient initially had meningitis for which he was started on ATT and, a few months later, had developed multiple intracranial tuberculomas with one in choroid plexus of leftlateral ventricle. Inspite of strict adherence to ATT, appearance of new lesion was an indication of biopsy. As endoscopy can solve dual purpose, biopsy and CSF diversion, it was chosen.

In conclusion, neurotuberculosis associated with ventricular lesions are associated with poor response to treatment and may need longer duration of treatment or other modes of effective drug delivery.
